# Lateral cephalometric analysis for treatment planning in orthodontics based on MRI compared with radiographs: A feasibility study in children and adolescents

**DOI:** 10.1371/journal.pone.0174524

**Published:** 2017-03-23

**Authors:** Alexander Heil, Eduardo Lazo Gonzalez, Tim Hilgenfeld, Philipp Kickingereder, Martin Bendszus, Sabine Heiland, Ann-Kathrin Ozga, Andreas Sommer, Christopher J. Lux, Sebastian Zingler

**Affiliations:** 1 Department of Neuroradiology, Heidelberg University Hospital, Heidelberg, Germany; 2 Division of Experimental Radiology, Department of Neuroradiology, Heidelberg University Hospital, Heidelberg, Germany; 3 Institute for Medical Biometry and Informatics, Heidelberg University Hospital, Heidelberg, Germany; 4 Department of Orthodontics and Dentofacial Orthopaedics, Heidelberg University Hospital, Germany; Julius-Maximilians-Universität Würzburg, GERMANY

## Abstract

**Objective:**

The objective of this prospective study was to evaluate whether magnetic resonance imaging (MRI) is equivalent to lateral cephalometric radiographs (LCR, “gold standard”) in cephalometric analysis.

**Methods:**

The applied MRI technique was optimized for short scanning time, high resolution, high contrast and geometric accuracy. Prior to orthodontic treatment, 20 patients (mean age ± SD, 13.95 years ± 5.34) received MRI and LCR. MRI datasets were postprocessed into lateral cephalograms. Cephalometric analysis was performed twice by two independent observers for both modalities with an interval of 4 weeks. Eight bilateral and 10 midsagittal landmarks were identified, and 24 widely used measurements (14 angles, 10 distances) were calculated. Statistical analysis was performed by using intraclass correlation coefficient (ICC), Bland-Altman analysis and two one-sided tests (TOST) within the predefined equivalence margin of ± 2°/mm.

**Results:**

Geometric accuracy of the MRI technique was confirmed by phantom measurements. Mean intraobserver ICC were 0.977/0.975 for MRI and 0.975/0.961 for LCR. Average interobserver ICC were 0.980 for MRI and 0.929 for LCR. Bland-Altman analysis showed high levels of agreement between the two modalities, bias range (mean ± SD) was -0.66 to 0.61 mm (0.06 ± 0.44) for distances and -1.33 to 1.14° (0.06 ± 0.71) for angles. Except for the interincisal angle (*p* = 0.17) all measurements were statistically equivalent (*p* < 0.05).

**Conclusions:**

This study demonstrates feasibility of orthodontic treatment planning without radiation exposure based on MRI. High-resolution isotropic MRI datasets can be transformed into lateral cephalograms allowing reliable measurements as applied in orthodontic routine with high concordance to the corresponding measurements on LCR.

## Introduction

Angular and linear measurements performed on lateral cephalometric radiographs (LCR) play a pivotal role in orthodontic routine diagnostics. Introduced in the 1930s [1] and further developed over many decades, lateral cephalometric analysis on LCR has remained the standard method in clinical routine until today. By assessing skeletal and dental relationships, it allows diagnosis and monitoring of various growth and development abnormalities [2]. For example, lateral cephalometric analysis is important for the evaluation of severe skeletal malocclusions and for the planning of orthodontic appliances or orthognathic surgery [2, 3]. Radiation protection is of major importance in orthodontics, as the vast majority of patients are children or adolescents and as in most cases a series of radiographs is taken in the course of treatment. Because of the increased lifetime risk for stochastic radiation effects [4–6], it would be desirable to perform imaging in complete absence of ionizing radiation.

As magnetic resonance imaging (MRI) is not associated with radiation exposure and capable to generate geometrically accurate datasets, it may evolve as a promising modality for cephalometric analysis as applied in orthodontics or related disciplines such as orthognathic surgery. Along with recent technical milestones, MRI is moving into focus in dental imaging [7]. Modern MRI methods can visualize dental and periodontal structures excellently due to increased field strength [8], parallel imaging techniques [9] and dedicated coil systems [10–12]. Reasons for the lack of MRI studies in orthodontics might be linked to specific requirements that have to be fulfilled to enable comprehensive and differentiated lateral cephalometric analysis. From the young patients’ perspective, examination time should be as short as possible and the procedure needs to be well-tolerated. Simultaneously, a large field of view is necessary to cover all relevant anatomic landmarks and the generated images must enable clear identification of dental as well as skeletal structures. Finally, image postprocessing should allow the performance of all established measurements required for treatment planning in correspondence to the measurements taken on LCR.

Here, we present an application-optimized, isotropic MRI technique that meets these criteria and a postprocessing algorithm that allows to transform the acquired MRI datasets into lateral cephalograms including the relevant midsagittal and bilateral landmarks. Based on this approach, a prospective *in vivo* study was performed to compare a series of well-established angular and linear measurements on LCR to those on corresponding MRI derived lateral cephalograms. The null hypothesis of non-equivalence was rejected if the measurements on LCR and MRI were within a low and clinically acceptable tolerance level of ± 2 mm and ± 2°, respectively. The purpose of the study was to evaluate whether MRI can be equivalent to LCR (“gold standard”) in cephalometric analysis.

## Materials and methods

### Ethics and funding

This prospective study was approved by the local research ethics committee of the University of Heidelberg (approval number: S-294/2014). Written informed consent was obtained from the patients, in case of minority from their parents as well.

### Patients

Twenty-one patients with various orthodontic disorders were enrolled in the study before treatment. Exclusion criteria were fixed orthodontic appliances, metal restorations, severe facial asymmetries, missing permanent incisors, no occlusion of either first premolars or second deciduous molars, contraindications to MRI and insufficient image quality of LCR or MRI. One patient had to be excluded because of head rotation around the vertical axis on LCR. Accordingly, 20 patients (8 females) were available for analysis. Mean age ± standard deviation was 13.95 years ± 5.34 (range, 8–26 years).

### Lateral cephalometric radiographs

All LCR were acquired using the imaging system Orthopos XG 3D^ready^ Ceph with a CCD line sensor (Sirona Dental Systems, Bensheim, Germany) at 72 kV, 15 mA, an exposure time of 9.4 s and a source-midsagittal plane distance of 1.5 m. Pixel size was 0.027 mm^2^. A 50 mm calibration ruler for magnification correction was integrated in the vertically aligned nose support of the device.

### MRI examinations

All MRI examinations were performed at a 3T MRI system (MAGNETOM Trio TIM; Siemens Healthcare, Erlangen, Germany) with a 16-channel multipurpose coil (Variety; Noras MRI products, Hoechberg, Germany). Apart from standard localizer sequences, a T1 weighted, isotropic SPACE (*sampling perfection with application optimized contrasts using different flip angle evolution*) sequence with an examination time of 6:59 min was conducted. This sequence included GRAPPA (*generalized autocalibrating partially parallel acquisitions*) for parallel imaging with an acceleration factor of 2, effective resolution was 0.68 mm^3^. Detailed sequence parameters are shown in Table 1. The field of view covered all relevant midsagittal and bilateral cephalometric landmarks. Prior to examination of study participants, the applied MRI technique was tested for geometric accuracy using the large ACR MRI Accreditation Phantom. According to the Phantom Test Guidance [13], seven measurements of known values were taken (1 end-to-end measurement with a known value of 148 mm, 6 diameter measurements each with a known value of 190 mm).

**Table 1:** Parameters of the used MRI sequence.

Sequence type	SPACE
Matrix	256 x 256
Field of view (mm x mm)	175 x 175
Section thickness (mm)	0.68
Voxel size (mm)	0.68 x 0.68 x 0.68
Number of sections	192
Repetition time (msec)	800
Echo time (msec)	26
Bandwidth (Hz/pixel)	501
Number of averages	2
Echo train length	63
Parallel imaging technique	GRAPPA (acceleration factor: 2)
Time of acquisition (min)	6:59

SPACE = sampling perfection with application optimized contrasts using different flip angle evolution, GRAPPA = generalized autocalibrating partially parallel acquisitions.

### Postprocessing of MRI datasets

**Fig 1:**
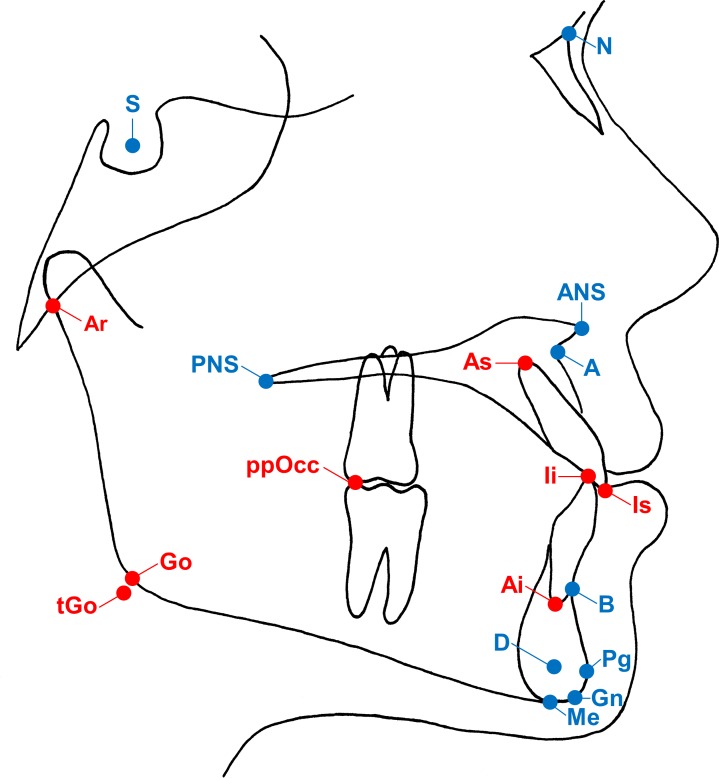
Cephalometric landmarks used in the present study. A total of 10 midsagittal (blue marked) and 8 bilateral (red marked) landmarks were included in cephalometric analysis: S = Sella; N = Nasion; ANS = Anterior nasal spine; PNS = Posterior nasal spine; A = Point A (most concave point of anterior maxilla); B = Point B (most concave point of mandibular symphysis); Is = Incision superius; Ii = Incision inferius; As = Apex superius; Ai = Apex inferius; Pg = Pogonion (most anterior point of mandibular symphysis); Gn = Gnathion (midpoint between Pg and Me); Me = Menton (most inferior point of mandibular symphysis); D = Point D (geometric center of the symphysis); Go = Gonion; tGo = Gonion tangent point (intersection between the mandibular line and the ramus line); Ar = (junction between inferior surface of the cranial base and the posterior border of the ascending rami of the mandible); ppOcc = posterior point of occlusion.

**Fig 2:**
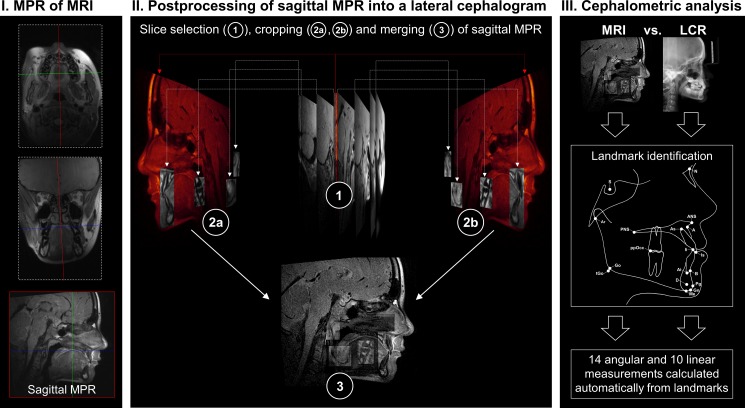
Workflow applied in the present study for each patient (n = 20) is shown. I = A multiplanar reconstruction (MPR) along the anatomic sagittal plane was acquired from primary magnetic resonance imaging (MRI) datasets. II = The midsagittal plane is coloured in red for better visualization of the workflow. Nine slices containing the landmarks necessary for cephalometric analysis were selected (1). The paired lateral slices were cropped preserving the relevant landmarks on the left (2a) and right (2b) side. The midsagittal plane and the 8 cropped lateral slices were merged into a lateral MRI cephalogram (3). III = Lateral cephalometric analysis was performed on lateral MRI cephalograms and corresponding lateral cephalometric radiographs (LCR) with dedicated software. For each modality two observers placed 10 midsagittal and 8 bilateral landmarks from which 14 angles and 10 distances were calculated automatically by software. Measurements were taken twice with an interval of 4 weeks.

Postprocessing of *in vivo* measurements was performed by two radiologists (ELG and AH, both radiology residents with 3 and 4 years of experience in dental imaging and image postprocessing, respectively). Multiplanar reconstructions (MPR) along the anatomic sagittal plane were acquired from primary MRI datasets. Sagittal MPR were transformed into lateral cephalograms covering the predefined landmarks (Fig 1) with dedicated software (AMIRA-3D v5.4.1; Zuse Institute, Berlin, Germany) as shown in Fig 2. Total time of postprocessing was approximately 15 minutes per patient.

### Cephalometric analysis of LCR and MRI

Lateral cephalometric analysis was performed on LCR and MRI cephalograms in DICOM format using dedicated software for cephalometry (Romexis v4.0.0; Planmeca, Helsinki, Finland). A customized analysis protocol with measurements widely used in orthodontic routine was predefined including Steiner’s analysis [14], the analysis module of the European board of Orthodontics [15] and Wits appraisal [16]. After calibration to the protocol, two independent observers (observer I: AH; observer II: SZ, an orthodontist with 8 years of experience in dental imaging) performed cephalometric analysis twice on each patient for both modalities with an interval of 4 weeks. Observers were blinded to the patients’ identities. All LCR were corrected for magnification with a known 50 mm distance on the calibration ruler. Eight bilateral and 10 midsagittal landmarks were traced (Fig 1). From these landmarks 14 angular and 10 linear measurements (Table 2) were performed automatically.

**Table 2:** Cephalometric measurements performed in the present study.

Measurement	Type	Definition
SNA	angular	Angle between lines SN and NA
SNB	angular	Angle between lines SN and NB
ANB	angular	Angle between lines AN and NB
SND	angular	Angle between lines SN and ND
SNPg	angular	Angle between lines SN and NPg
SN/ML	angular	Angle between SN line and mandibular line (Me-tGo)
NL/ML	angular	Angle between nasal line (ANS-PNS) and mandibular line (Me-tGo)
SN/OcP	angular	Angle between SN line and occlusal plane (OcP)*
SN/GoGn	angular	Angle between lines SN and GoGn
Ar-Go-Me	angular	Angle between lines Ar-Go and Go-Me (“gonial angle”)
Ui/Li	angular	Angle between lines through long axis of upper and lower central incisors
Ui/SN	angular	Angle between line through long axis of upper central incisor and SN line
Ui/NA	angular	Angle between line through long axis of upper central incisor and NA line
Li/NB	angular	Angle between line through long axis of lower central incisor and NB line
Wits	linear	Measurement of perpendicular projection of points A and B to occlusal plane
Pg/NB	linear	Distance between point Pg and NB line
A/NPg	linear	Distance between point A and NPg line
S-Go	linear	Distance between points S and Go (“posterior facial height”)
N-Me	linear	Distance between points N and Me (“anterior facial height”)
Ii/NB	linear	Distance between incision inferius and NB line
Is/NA	linear	Distance between incision superius and NA line
Overjet	linear	Horizontal distance between tips of upper and lower central incisors
Overbite	linear	Vertical distance between tips of upper and lower central incisors
S-Go/N-Me	linear (ratio)	Ratio of distance S-Go to distance N-Me

Abbreviations for cephalometric landmarks are explained in the footnote of Fig 1.

* OcP was defined as the line passing through the midpoint between the incisal edges (anterior) and the most distal point of contact of either the first permanent or second deciduous molars (posterior).

### Statistical analysis

Statistical analysis was performed with software (R version 3.3.1; R Foundation for Statistical Computing, Vienna, Austria). For all measurements, intra- and interobserver agreement was analyzed by intraclass correlation coefficient (ICC). Bland-Altman analysis was used to assess the agreement between the two modalities [17] for each type of measurement with average values of the two time points and two investigators. Statistical analysis aimed to test for equivalence between the corresponding measurements on LCR and MRI. In this approach, equivalence can be claimed when the confidence interval of the difference in outcome between the compared groups is within a predetermined equivalence margin that can be justified clinically and scientifically [18]. Equivalence testing between LCR and MRI was carried by two one-sided tests (TOST) with α = 0.05 and a 1─2α confidence interval [19], also using average values of the two time points and two investigators. Prior to testing, equivalence margins (± θ) of ± 2 mm and ± 2° were defined, referring to clinically acceptable levels of variance for lateral cephalometric analysis as published before [20, 21]. Null hypothesis of TOST was that the two mean values were not equivalent. If the 1–2α confidence interval was completely contained within the ± θ interval, the null hypothesis was rejected and the two datasets were considered equivalent (*p-*value < 0.05).

## Results

According to the ACR Phantom Test Guidance [13], all seven measurements performed with the MRI sequence used in the study (Table 1) were congruent with the known values of the ACR Phantom.

Both observers showed very high intraobserver agreement for MRI measurements, average (± SD, range) intraobserver ICC were 0.977 (± 0.019, 0.926–0.996) for observer I and 0.975 (± 0.017, 0.937–0.992) for observer II. Similar intraobserver ICC were observed for the LCR counterparts with mean values (± SD, range) of 0.975 (± 0.016, 0.935–0.997) for observer I and 0.961 (± 0.065, 0.692–0.998) for observer II.

Interobserver agreement was excellent for MRI with an average (± SD, range) ICC of 0.980 (± 0.014, 0.938–0.997). In comparison, interobserver agreement for LCR was also excellent, but moderately lower compared to MRI with an average (± SD, range) ICC of 0.929 (± 0.106, 0.467–0.996). Intraobserver and interobserver ICC for all measurements are shown in Table 3.

**Table 3:** Interobserver and intraobserver agreement for lateral cephalometric measurements.

Measurement	Interobserver ICC		Intraobserver ICC	
	LCR	MRI	LCR (Obs. I; Obs. II)	MRI (Obs. I; Obs. II)
SNA [°]	0.935	0.979	0.966; 0.959	0.965; 0.975
SNB [°]	0.968	0.993	0.989; 0.992	0.988; 0.989
ANB [°]	0.958	0.984	0.987; 0.987	0.978; 0.980
SND [°]	0.960	0.994	0.987; 0.991	0.985; 0.992
SNPg [°]	0.968	0.995	0.989; 0.992	0.993; 0.990
SN/ML [°]	0.984	0.991	0.990; 0.995	0.985; 0.985
NL/ML [°]	0.970	0.988	0.983; 0.993	0.992; 0.981
SN/OcP [°]	0.916	0.978	0.935; 0.974	0.970; 0.937
SN/GoGn [°]	0.970	0.984	0.965; 0.991	0.982; 0.985
Ar-Go-Me [°]	0.975	0.977	0.966; 0.980	0.980; 0.983
Ui/Li [°]	0.984	0.982	0.961; 0.984	0.991; 0.992
Ui/SN [°]	0.964	0.977	0.964; 0.975	0.991; 0.983
Ui/NA [°]	0.977	0.970	0.967; 0.977	0.989; 0.977
Li/NB [°]	0.911	0.983	0.958; 0.912	0.986; 0.978
Wits [mm]	0.870	0.975	0.972; 0.979	0.978; 0.963
Pg/NB [mm]	0.894	0.963	0.946; 0.990	0.937; 0.938
A/NPg [mm]	0.965	0.984	0.983; 0.989	0.978; 0.978
S-Go [mm]	0.985	0.993	0.992; 0.992	0.992; 0.978
N-Me [mm]	0.996	0.997	0.997; 0.998	0.996; 0.991
Ii/NB [mm]	0.923	0.980	0.986; 0.926	0.979; 0.978
Is/NA [mm]	0.941	0.938	0.977; 0.923	0.938; 0.958
Overjet [mm]	0.467	0.974	0.984; 0.692	0.959; 0.961
Overbite [mm]	0.854	0.958	0.977; 0.884	0.926; 0.942
S-Go:N-Me [mm/mm]	0.964	0.992	0.988; 0.991	0.981; 0.978
**mean**	**0.929**	**0.980**	**0.975; 0.961**	**0.977; 0.975**

ICC = Intraclass correlation coefficient; Obs. = Observer

Abbreviations for cephalometric landmarks are explained in the footnote of Fig 1.

**Fig 3:**
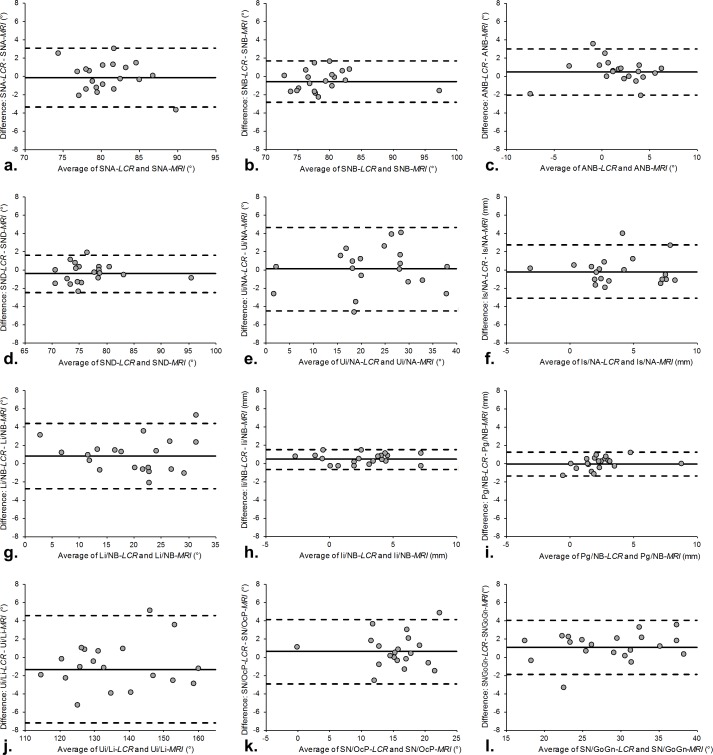
Bland-Altman plots show the differences between the measurements on LCR and lateral MRI cephalograms. Solid lines represent the mean of all differences (bias), dashed lines represent the 95% limits of agreement. Exemplary measurements according to Steiner’s analysis [14] are shown in this figure: (a) SNA-angle, (b) SNB-angle, (c) ANB-angle, (d) SND-angle, (e) Ui/NA-angle, (f) Is/NA-distance, (g) Li/NB-angle, (h) Ii/NB-distance, (i) Pg/NB-distance, (j) Ui/Li-angle, (k) SN/OcP-angle and (l) SN/GoGn-angle.

Bland-Altman analysis revealed high of levels agreement between the two modalities for all measurements, bias range (mean ± SD) was -0.66 to 0.61 mm (0.06 ± 0.44) for linear and -1.33 to 1.14° (0.06 ± 0.71) for angular measurements (Table 4). Exemplary Bland-Altman plots according to Steiner’s analysis [14] are shown in Fig 3. At the predefined equivalence margins (± θ) of ± 2 mm / ± 2° statistical equivalence between MRI and LCR was observed in 23 out of 24 measurements (*p* < 0.05), only for the interincisal angle (Ui/Li) the null hypothesis of non-equivalence could not be rejected (p = 0.17) (Table 4). This result is in line with the corresponding Bland-Altman analysis, where Ui/Li showed the highest bias (-1.33°) and the widest 95% limits of agreement (-7.22°, 4.56°) of all measurements (Table 4, Fig 3).

**Table 4:** Lateral cephalometric measurements from LCR and MRI (n = 20).

Measurement	LCR^a^			MRI^a^			Mean difference (LCR—MRI)	95% limits of agreement (LCR—MRI)	*p-*value^b^
SNA [°]	80.95	±	3.59	81.08	±	3.98	-0.13	-3.32, 3.06	2,95 x 10^−5^
SNB [°]	79.03	±	5.16	79.60	±	5.15	-0.57	-2.81, 1.67	1.11 x 10^−5^
ANB [°]	1.92	±	3.22	1.48	±	3.24	0.44	-2.08, 2.96	1.55 x 10^−5^
SND [°]	76.75	±	5.46	77.19	±	5.46	-0.44	-2.46, 1.57	9.34 x 10^−7^
SNPg [°]	80.40	±	5.37	81.01	±	5.33	-0.61	-2.56, 1.33	2.65 x 10^−6^
SN/ML [°]	31.36	±	6.74	30.22	±	6.42	1.14	-1.71, 3.98	7.78 x 10^−3^
NL/ML [°]	26.32	±	7.02	26.05	±	6.98	0.27	-3.95, 4.48	9.41 x 10^−4^
SN/OcP [°]	15.77	±	4.93	15.17	±	4.87	0.60	-2.93, 4.12	1.21 x 10^−3^
SN/GoGn [°]	29.06	±	6.44	27.97	±	6.09	1.09	-1.89, 4.08	7.81 x 10^−3^
Ar-Go-Me [°]	126.52	±	6.04	126.95	±	5.66	-0.43	-2.50, 1.64	1.16 x 10^−6^
Ui/Li [°]	134.92	±	13.98	136.25	±	13.27	-1.33	-7.22, 4.56	0.17
Ui/SN [°]	103.82	±	9.40	103.86	±	9.69	-0.04	-4.14, 4.05	2.50 x 10^−4^
Ui/NA [°]	22.87	±	9.93	22.78	±	9.65	0.09	-4.48, 4.65	8.13 x 10^−4^
Li/NB [°]	20.29	±	7.95	19.49	±	7.96	0.80	-2.80, 4.41	4.51 x 10^−3^
Wits [mm]	-1.10	±	3.96	-1.50	±	3.68	0.40	-2.07, 2.87	8.98 x 10^−6^
Pg/NB [mm]	2.41	±	2.06	2.46	±	1.83	-0.05	-1.37, 1.27	3.60 x 10^−11^
A/NPg [mm]	0.60	±	3.15	0.18	±	3.22	0.42	-2.26, 3.00	1.76 x 10^−5^
S-Go [mm]	71.23	±	6.67	71.83	±	6.67	-0.60	-3.73, 2.54	4.53 x 10^−4^
N-Me [mm]	107.36	±	9.63	107.15	±	9.10	0.21	-2.72, 3.14	1.83 x 10^−5^
Ii/NB [mm]	2.83	±	2.63	2.41	±	2.65	0.42	-0.70, 1.54	7.84 x 10^−11^
Is/NA [mm]	3.82	±	3.07	4.04	±	3.08	-0.22	-3.14, 2.70	1.89 x 10^−5^
Overjet [mm]	3.77	±	2.60	3.82	±	2.52	-0.05	-1.70, 1.60	1.50 x 10^−9^
Overbite [mm]	2.72	±	2.21	2.11	±	2.45	0.61	-1.40, 2.62	3.91 x 10^−6^
S-Go/N-Me [mm/mm]	66.53	±	5.52	67.19	±	5.55	-0.66	-3.63, 2.30	4.24 x 10^−4^

^a^ Values are means ± standard deviations of measurements of two time points and two investigators.

^b^ All *p*-values refer to two one-sided tests (TOST) of equivalence with predefined equivalence margins (± θ) of ± 2° and ± 2 mm, respectively. *p-*values < 0.05 indicate statistical equivalence.

## Discussion

In particular in children and adolescents, avoidance of radiation exposure is crucial. In this study, we aimed to show equivalence of MRI to radiographs in lateral cephalometry as a basis for orthodontic treatment planning. To our knowledge, MRI based standardized lateral cephalometric analysis including midsagittal as well as bilateral landmarks has not been evaluated before. An isotropic T1-weighted sequence with excellent contrast, high spatial resolution and short scanning time formed the basis for our new approach. Images yielded from this MRI technique allowed a clear depiction of the dental and skeletal cephalometric landmarks. The subsequent postprocessing algorithm enabled the transformation of the isotropic MRI datasets into lateral cephalograms covering the midsagittal and bilateral landmarks necessary for diagnostics and treatment planning in orthodontics. Based on these generated lateral MRI cephalograms it was possible to perform a detailed cephalometric analysis with a broad spectrum of measurements as used in orthodontic routine. Linear and angular cephalometric measurements taken on lateral MRI cephalograms turned out to be highly reliable as interobserver and intraobserver agreement was excellent. As a principal finding, we found high levels of agreement between the measurements on lateral MRI cephalograms and the corresponding measurements on LCR in a clinical environment by examining young patients with various orthodontic abnormalities. Statistical equivalence between the two modalities was shown for 23 out of 24 measured distances and angles within a strict predefined equivalence margin of ± 2 mm / ± 2°. The only measurement without statistical equivalence was the interincisal angle, which also showed a slightly higher bias level in Bland-Altman analysis in comparison to the other cephalometric measurements. This, however, was not an unexpected finding, as the interincisal angle is prone to measurement errors when performed on LCR [22, 23]. Nonetheless, the mean difference of -1.33° in Bland-Altman analysis still indicated a low and clinically tolerable bias for the interincisal angle. Considering the overall high concordance with LCR (“gold standard”) and the absence of radiation exposure, lateral cephalometric analysis for the assessment and monitoring of orthodontic conditions could be performed by MRI in the future to keep radiation dose in young patients as low as possible.

Even though mean differences between LCR and MRI were generally low, they should be analyzed thoroughly. As intra- and interobserver reliability were consistently high for both modalities, the slight differences were presumably due to systematic errors. Like all radiographic techniques, LCR are accompanied by distortion and magnification [22, 24–26]. As we proved geometric accuracy for the applied MRI technique by standardized phantom measurements, it is most likely that the slight differences for angular and linear measurements predominantly derived from LCR. Considering that studies comparing conventional computed tomography (CT) or cone-beam computed tomography (CBCT) to LCR showed very similar differences in lateral cephalometric measurements [21, 27, 28] and that CT-techniques are geometrically accurate under normal conditions [29], it is legitimate to compare these results to ours. The hypothesis that intrinsic limitations of LCR were the main error source in the present study is strongly supported by *ex vivo* studies, which showed very high concordance between measurements on MRI and CT [30] or MRI and CBCT [31] [32].

An essential element of our feasibility study was a MRI technique with the potential to become a routine application for orthodontic treatment planning. It should be highlighted that we were able to provide a short protocol which was well-tolerated by the children and adolescents who participated in the study. Including patient positioning and planning on standard localizer sequences, the MRI examinations were performed within a total time of about 10 minutes leading to high compliance without relevant motion artifacts.

Our study aimed to compare MRI with LCR due to high relevance in orthodontic routine. However, potential capabilities of the applied MRI technique are not restricted to lateral cephalometry. The second important radiographic image tool in orthodontics are panoramic radiographs (PR), typically used for evaluation of dental development, unerupted or supernumerary teeth and alveolar bone morphology [33]. As of principle, such analyses are also feasible on MRI datasets as acquired in our study. If future studies showed equivalence between MRI and PR, the latter could be avoided providing the possibility of orthodontic imaging without any radiation exposure. Furthermore, isotropic MRI datasets have the potential to perform three-dimensional (3D) cephalometric analysis, which might lead to more differentiated and conclusive diagnoses compared to two-dimensional radiographs. Several approaches for 3D cephalometry have been made based on CT and CBCT, but reliable procedures could not be established due to the lack of comparative norms [34]. By contrast, non-ionizing MRI provides the possibility to establish proper standards of 3D cephalometry, as the whole spectrum of orthodontic conditions including normal collectives and patients with slight malconditions could be analyzed. Another advantage of MRI over X-ray methods is the visualization of soft tissues. This a key point for future studies, as there are no objective methods to monitor changes in soft tissues under therapy [35].

A limitation of the present study was that the true values of the cephalometric measurements were not known. Even though lateral cephalometry on LCR is the “gold standard”, it is prone to measurement errors as described above and therefore should not be used as a reference standard in a diagnostic accuracy study. Thus, accuracy for MRI can only be claimed for the phantom measurements, but not for *in vivo* data.

A further limitation was that MRI datasets had to be postprocessed to generate the lateral cephalograms necessary for data analysis. Specific postprocessing software was required and the algorithm could only be performed with sufficient user experience. However, this limitation is not surprising regarding the framework of a feasibility study aiming at introducing this new approach of MRI based cephalometric analysis. As a next step, we suggest the implementation of software solutions allowing user-friendly and time-efficient postprocessing of primary MRI datasets into lateral cephalograms. Ideally, only sagittal MPR and selection of slices with the relevant landmarks will have to be performed by the user in such applications. All subsequent steps to the final lateral cephalogram could then be computed fully automated without user interaction. Furthermore, future software for MRI based cephalometric analysis should be integrated into existing standard software to facilitate broad application in clinical routine.

## Conclusions

In conclusion, this study shows that full lateral cephalometric analysis as applied in orthodontics is feasible based on postprocessed MRI datasets. There was a high concordance with equivalent measurements taken on LCR, which is the standard method in clinical routine. Our MRI based approach for the first time enables the assessment of orthodontic conditions by using clinically standardized analysis methods in absence of radiation exposure to the mostly young patients. The short and well-tolerated examination protocol applied in our feasibility study could be integrated into clinical routine. Further studies with large patient populations using different MRI systems should be conducted to support our findings and to evaluate whether MRI and LCR are equivalent in lateral cephalometric analysis under the most diverse clinical and technical conditions. Moreover, our MRI technique has the potential to overcome the limitations of projection radiography in the future.
